# Influence of intellectual disability on exercise regulation: exploring verbal, auditory and visual guidance to contribute to promote inclusive exercise environments

**DOI:** 10.1136/bmjsem-2023-001765

**Published:** 2024-01-05

**Authors:** Kandianos Emmanouil Sakalidis, Stein Gerrit Paul Menting, Florentina Johanna Hettinga

**Affiliations:** 1 Sport Exercise and Rehabilitation, Northumbria University, Newcastle upon Tyne, UK; 2 School of Psychology, Ulster University, Coleraine, UK

**Keywords:** Exercise, Disability, Energy expenditure, Sport and exercise psychology

## Abstract

**Objective:**

The role of intellectual disability (ID) in exercise regulation has remained largely unexplored, yet recent studies have indicated cognitive-related impaired pacing skills in people with ID. In a well-controlled laboratory environment, this study aims to (1) establish the role of ID in pacing and explore the ability of people with and without ID to maintain a steady pace; (2) to investigate if verbal feedback and/or (3) the presence of a pacer can improve the ability of people with ID to maintain a preplanned submaximal velocity.

**Methods:**

Participants with (n=10) and without ID (n=10) were recruited and performed 7 min submaximal trials on a cycle ergometer (Velotron). Participants with ID also performed a cycling trial with a pacer (virtual avatar).

**Results:**

The non-parametric tests for repeated measures data (p≤0.05) showed that (1) people with ID deviated more from the targeted pace compared with people without ID, (2) the verbal feedback did not influence their ability to keep a steady pace and (3) they deviated less from the targeted pace when a visual pacer was introduced.

**Conclusion:**

The results revealed the difficulties of people with ID in planning and monitoring their exercise and the difficulties in appropriately responding to auditory and verbal feedback. Coaches and stakeholders who want to offer inclusive exercise pathways should consider that people with ID perform and pace themselves better when supported by intuitive, visual and personally meaningful stimuli such as other cyclists (avatars).

**WHAT IS ALREADY KNOWN ON THIS TOPIC**

- Pacing is a self-regulatory process for fatigue management, the management of affective responses and optimal performance during exercise.
- The ability to regulate exercise intensity differs between people with and without intellectual disabilities (ID).

**WHAT THIS STUDY ADDS**

- People with ID have an impaired ability to maintain a preplanned submaximal cycling velocity compared with people without ID.
- Verbal feedback does not improve the ability of people with ID to maintain a preplanned submaximal cycling velocity.
- The presence of a pacer (visual feedback) significantly improves the ability of people with ID to maintain a preplanned submaximal cycling velocity.

**HOW THIS STUDY MIGHT AFFECT RESEARCH, PRACTICE OR POLICY**

- Knowledge gained from this study can be used by coaches who want to effectively coach people with ID and stakeholders who want to offer inclusive exercise opportunities and facilitate and increase physical activity in people with ID.

## Introduction

Despite the psychological, physiological and social benefits of an active lifestyle,^1^ only a small number (9%) of people with intellectual disability (ID) regularly participate in exercise and sports activities.^2^ According to the International Classification of Functioning, Disability and Health (ICF), which provides a framework for understanding health and disability, their inactive lifestyle could be caused and further amplified due to the specific ‘functions’ of this population,^3^ like inadequate exercise regulation and pacing behaviour.^4 5^ This results in unnecessary fatigue and discomfort, with higher exercise intensities associated with negative affective responses.^6^ Pacing is the self-regulatory decision-making process in which people must decide how and when to distribute their energy resources during exercise.^7 8^ It is an influential factor in exercise regulation and behaviour in people with chronic conditions and disabilities^9 10^ and essential for optimal sports performance in athletes.^11 12^

A limited number of studies investigating the pacing behaviour of people with ID in sports and exercise^4 5 13^ have shown that athletes with ID deviate more from a targeted pace during a submaximal running trial^4^ and their competitive pacing behaviour differed compared with athletes without ID.^5 13^ This is suggested to occur due to the deficits that people with ID experience in self-regulatory skills involved in pacing, such as goal setting, self-monitoring, self-control and self-reactions (‘functions’, according to the ICF).^3 14 15^ Thus, to optimise energy regulation, it is important to explore how the contextual factors (eg, environmental) could facilitate the self-regulation of the pacing behaviour of people with ID.^16^

Environmental interventions, such as social support and guidance, as well as the attitudes of other people, could potentially support exercise activities for people with ID (eg, cycling) and enhance their active, long-term participation in sports settings.^3^ Although providing auditory feedback appears to be a useful strategy for guiding exercise and motor activities,^17^ its potential role in supporting the regulation of pacing behaviour has not been investigated. Also, the social environment (eg, peers, coaches and other exercisers) could play an important role in people’s pacing and self-regulatory skills.^15 18 19^ It can act as a social placebo, motivate exercisers and athletes to set goals and plan their actions, provide visual guidance, thus direct pacing and performance feedback, improve people’s attention and positively influence their affective reactions.^15 18 19^ Moreover, when people are less proficient at the self-regulatory process of pacing (as people with ID are), coaches can use the environment to facilitate their self-regulatory and pacing skills acquisition.^15 16 20^ For instance, coaches’ verbal feedback and guidance could alter athletes’ activity monitoring and facilitate their self-control during activity.^15 20^ At the same time, the introduction of visual stimuli, such as avatars or opponents, has been shown to impact performance positively,^21^ providing meaningful social invitations for action through direct perception of certain cues.^21^ For instance, Konings *et al*^22^ showed that the presence and behaviour of an opponent affect pacing-related decisions in maximal cycling trials. However, it would be interesting to explore the influence of contextual factors, like the social environment, in different, less sports-performance-oriented situations with an impact on training and exercise scenarios.

In addition to this, previous studies revealed that athletes with ID engage mainly in intuitive, less cognitively demanding decision-making contexts.^13^ Thus, different perceptual affordances will likely affect their pacing behaviour in a constantly changing sports environment, which invites them to adapt and continuously alter their pacing behaviour and physical activity-related actions.^7 13 21^ That means other exercisers/athletes (eg, pacers) could act as affordances, provide intuitive, visual guidance during exercise tasks to people with ID and facilitate self-regulatory behaviour.^7 13 21^ However, to date, only one study has investigated the influence of other exercisers on the pacing behaviour of people with ID, and it focused solely on maximal trials and sports performance rather than on submaximal exercise and pacers, which is more relevant to training and physical activity engagement.^23^

Based on all the above, and with the purpose of better understanding the role of ID in exercise regulation, this paper explores the differences in the ability of people with and without ID to maintain a preplanned steady pace (a critical aspect of pacing) in a well-controlled laboratory environment. As we hypothesise that people with ID will have an impaired ability to maintain a preplanned submaximal cycling velocity, confirming the field study of van Biesen *et al*,^4^ exploring strategies to support their exercise regulation is critical. This paper also investigates if verbal feedback could facilitate the ability of people with ID to maintain a submaximal velocity and explores if visual (guidance from an avatar/pacer) or auditory feedback (cycling alone) can help participants with ID to keep a steady pace during a cycling activity. We hypothesise that these contextual factors (verbal feedback and, mainly, the visual feedback from a social stimulus) can help to improve pacing behaviour and energy regulation in submaximal exercise.^4 15^ The findings of this paper could help to better understand the complex interactions among people’s ‘health condition’ (ID), ‘functions’ (self-regulatory and pacing deficits) and ‘contextual factors’ (auditory and social feedback) through the ICF framework.^3^ A better understanding of these interactions could lead to the promotion of more appropriate exercise activities and the endorsement of inclusive exercise and sport participation for all.

## Methods

### Participants

Participants were recruited through sports clubs, charities and sports organisations via emails and phone calls (from January 2020 to April 2022). An a priori power analysis (power=0.80, significance criterion α=0.05) was conducted by the researchers using G*Power V.3.1.9.7 to determine the sample size.^24^ Thus, 10 participants (2 females and 8t males; mean age=37, SD=7 years) and another 10 participants without ID (4 females and 6 males; mean age=24, SD=2 years) consented to participate in our study. All participants gave their consent (verbal and written) before they participated in the research activity.

None of the participants had any previous experience in time trial cycling. All the participants with ID met the criteria for diagnosis of ID as set by the British Psychological Society^14^: limitations in intellectual and adaptive functioning with an IQ≤70, limitations in practical, social and conceptual skills, and manifested before the age of 18 years old. Before the testing, participants completed the Physical Active Readiness Questionnaire+.^25–27^ Participants with severe ID and/or participants who could not understand the concept of activities were excluded from the study. Participants with physical impairments or any related chronic conditions that may be contraindicated or exacerbated by cycling were also excluded. The International Physical Activity Questionnaire-Short Form, a valid and reliable measure that estimated participants’ physical activity levels,^28^ was used to recruit moderate to highly active participants with and without ID.

### Patient and public involvement

Patients/participants, nor the public, were involved in the design or dissemination of this study. We aim to publish open access to reach a wide group of stakeholders, mostly targeting professionals working with participants with ID.

### Equity, diversity and inclusion statement

Our authors’ team comprises two early-career male researchers (first and second author) and one female (senior and corresponding author) of different nationalities and backgrounds. The participants included in this study represent a spectrum of ages, demographics and disabilities, including males and females with and without ID.

### Procedure

All participants visited the laboratory on three separate occasions and performed a 7 min submaximal cycling trial per visit (three trials with at least 1-week difference between each visit). The goal of the cycling trials was to maintain a preplanned, submaximal velocity for 7 min. All the trials were performed on the Velotron cycle ergometer using the Velotron 3D software (Velotron Dynafit, Racermate, Seattle, USA). A straight, flat course with no wind and with white markers every 75 m was selected. A projector was used so participants could see their virtual avatar on a projection screen during the trials. The purpose of the familiarisation visit (visit 1) was for participants to understand the protocol and to get familiar with the equipment and the submaximal cycling trial. After completing the submaximal cycling trial, participants performed a 4 km cycling time trial at maximal effort. During visits 2 and 3, the targeted velocity was set to 70% of the mean velocity reached on the 4 km cycling time trial during the familiarisation visit. This targeted velocity was chosen so the participants could feasibly keep a steady pace without experiencing notable fatigue, a state that deteriorates pacing.^12^ The researchers kept track of participants’ velocity, power output and distance covered during all the trials (25 Hz). Researchers took safety precautions and measures to prevent unnecessary injuries and illness (COVID-19 related) and thoroughly explained them to the participants. All trials were conducted at ambient temperature levels, between 19°C and 21°C.

**Figure 1:**
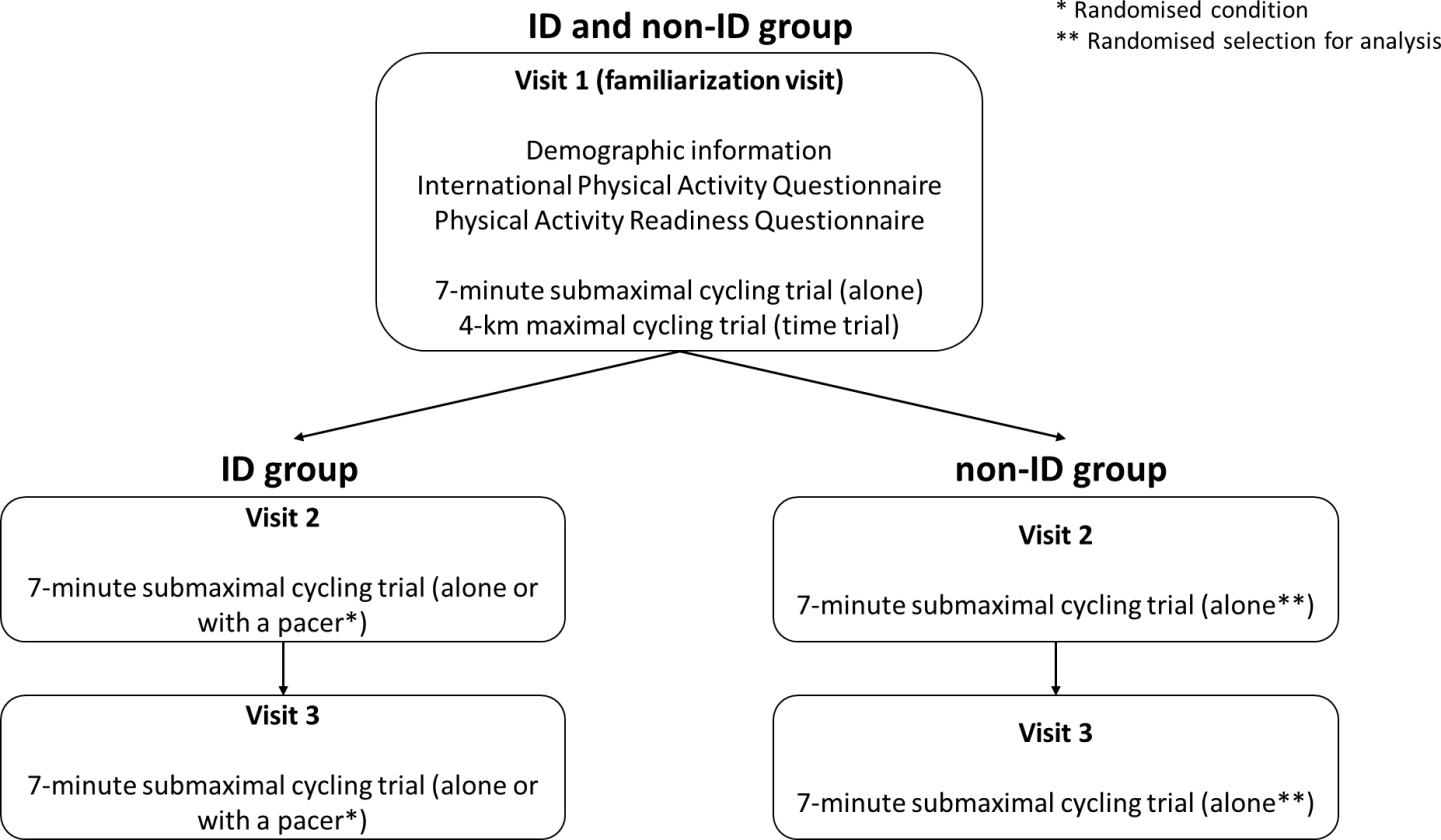
Flow chart of the research protocol for the ID and the non-ID group. ID, intellectual disability.

Participants without ID participated only in one condition, without another pacer (alone). Participants with ID participated in two different, randomised conditions, alone and with a real-life-sized pacer (virtual avatar). For all the conditions, participants first reached their targeted (preplanned) velocity based on researchers’ standardised velocity-related feedback (eg, please go faster, please go slower, keep that speed) and then the trials began. For the condition without the pacer (alone) but with auditory feedback, audio speakers were used so participants could hear an auditory signal whenever they reached a 75 m marker. Hearing the auditory signal before reaching the 75 m marker implies that the participants needed to cycle faster. Hearing the auditory signal after passing the 75 m marker implies that the participants needed to cycle slower. For the condition with a pacer, a second virtual avatar was introduced who was cycling with a set, targeted velocity (70% of the mean velocity that the participant reached on the 4 km cycling time trial during the familiarisation visit), next to the participant’s avatar. Participants with ID were instructed to always cycle alongside the second avatar. Cycling in front of the pacer implies that the participants need to cycle slower. Cycling behind the pacer implies that the participants need to cycle faster. In all trials, researchers gave standardised, speed-related feedback during the first 5 min. During the last 2 min of the trial, feedback was not provided. A computerised randomisation using Excel (Microsoft Office Excel, 2007) was used to determine (1) the condition that each participant with ID participated in each visit and (2) the visit (2 or 3) that the researchers should take into consideration in the analysis for each participant without ID (for the research protocol flow chart, please see figure 1).

### Statistical analysis

Due to the violation of normality assumption using a Shapiro-Wilk test of normality, a series of rank-based non-parametric analyses were conducted. For all the analyses, the velocity (provides direction from the targeted velocity) and the absolute percentage deviation (APD; the percentage deviation from the targeted velocity without considering the direction of the deviation) were used as the main dependent variables.

To test the differences in the ability of people with and without ID to maintain a preplanned steady pace (aim 1), we performed non-parametric tests for repeated measures data. More specifically, the Anova-Type Statistic (ATS) and the modified ATS were used to test main and interaction effects.^29–31^ For the first aim’s analyses, we used the minutes as the within-subjects factor (seven time points). Participants’ and targeted velocities for each group were used as the between-subjects factors for the velocity differences. In contrast, for the APD differences, the group (ID and non-ID group) was used.

To test whether verbal feedback could facilitate the ability of people with ID to maintain a submaximal velocity (aim 2), we performed a series of ATS per condition (for the ‘alone’ and the ‘pacer’ condition) with the minutes as the within-subjects factor (seven time points). Multiple stepwise procedures confirmed if there were any significant velocity and/or APD differences between the first five (with feedback) and the sixth or seventh minute (without feedback) within each trial (with an adjusted p value based on Campbell and Skillings modification).

To explore if the introduction of a pacer can help participants with ID to keep a steady pace (aim 3), we performed another ATS for each dependent variable. For the third aim’s analyses, we used the minutes as the within-subjects factor (seven time points). Participants’ velocity (in both conditions) and targeted velocity were used as the between-subjects factors for all the velocity differences. In contrast, for the APD differences, the condition (‘alone’ and ‘pacer’) was used.

If any ATS yielded significant differences (between-factor main effects and/or interaction effects), pairwise comparisons between groups and/or conditions were performed at each time point (with a Bonferroni-adjusted p value). The statistical analyses above used the ‘nparLD’ and ‘nparcomp’ functions in R.^29–32^ Using these functions, the relative effects were also calculated (an increase in the effect indicates an increase in the measured conditions/groups/distances).^29–32^ In addition to all the above, the IQR and the non-parametric coefficient of variation (np-CV) explored the velocity variation of each group (per minute). Moreover, the Mann-Whitney U test explored the IQR and the np-CV differences between the groups, while a Wilcoxon signed-rank test explored the IQR and the np-CV differences between the conditions of the ID group. All the analyses were performed by using R V.4.1.1, and the significance level was set at p≤0.05.

## Results

**Figure 2:**
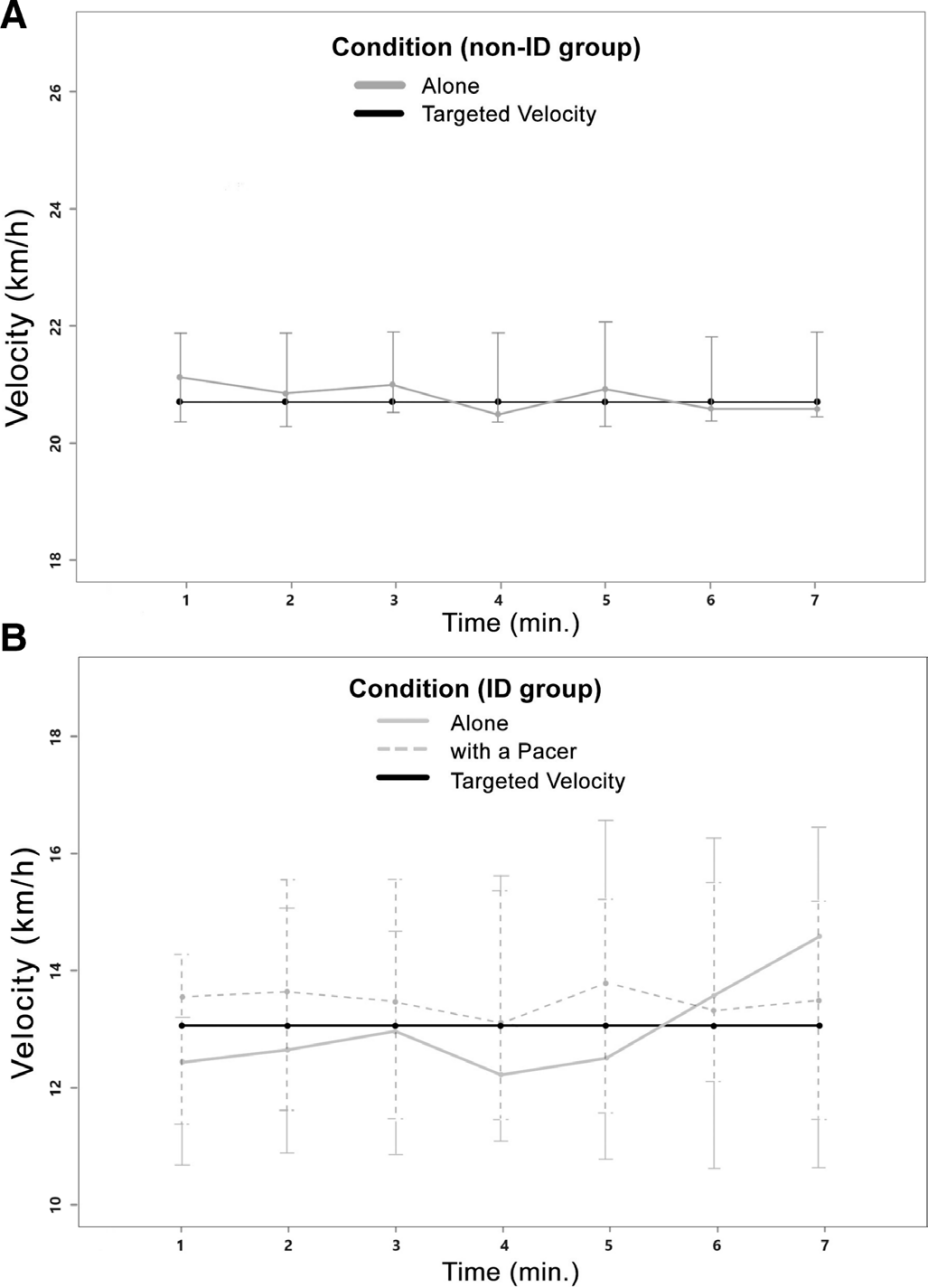
Line graphs with the participants’ median velocity and targeted velocity (+IQRs) for the non-ID group (A) and the ID group (B). ID, intellectual disability.

The first aim’s analyses (investigating differences in the ability of people with and without ID to maintain a preplanned steady pace) did not reveal significant (main nor interaction effects) differences between the participants’ velocity and the targeted velocity in ID or the non-ID group. However, the velocity’s IQR and the np-CV were significantly higher in participants with ID (p<0.001 for both variables; see table 1 and figure 2 for the velocity variability per minute of the non-ID and the ID group). The APD was significantly higher in participants compared with participants without ID, F(1, 13.13) **=** 64.87, p<0.001 (between-factor main effect). The multiple comparisons revealed significant differences between groups (p<0.001 for the first 5 min and p=0.01 for the seventh minute) at each time point of the trial except for the sixth minute (p=0.16). In both groups, the APD did not significantly change throughout the trial (see table 2 for the descriptive statistics and figure 2 for the participants’ velocity deviation from the targeted velocity for the non-ID and the ID group).

**Table 1:** Velocity variability of the non-ID group and the ID group in both conditions (‘alone’ and ‘with a pacer’)

Variable	Group	Condition	Minutes						
Minutes			1	2	3	4	5	6	7
np-CV (%)									
	Non-ID	Alone	0.95	5.03	2.08	4.20	0.76	1.52	1.80
	ID	Alone	20.59	22.24	20.46	18.85	23.50	28.34	33.51
	ID	Pacer	15.56	20.33	20.90	18.02	15.78	19.04	15.40

ID, intellectual disability; np-CV (%), non-parametric coefficient of variation (%).

**Table 2:** Descriptive statistics of absolute percentage deviation (APD) (%), velocity (km/hour) and targeted velocity (km/hour) variables for ID and non-ID groups in both conditions (alone and with a pacer) during the 7 min trials

Variable	Source	Mdn	IQR	Min	Max
APD					
	ID group (alone)	11.16	32.14	0.13	74.41
	ID group (pacer)	2.92	7.31	0.08	37.62
	Non-ID group (alone)	0.90	0.89	0.08	2.31
Velocity					
	ID group (alone)	12.62	5.08	8.02	21.83
	ID group (pacer)	13.42	3.46	8.84	16.29
	Non-ID group (alone)	20.85	1.67	18.53	24.15
Targeted velocity					
	ID group	13.00	2.50	9.40	15.40
	Non-ID group	20.80	1.30	18.60	23.60

ID, intellectual disability; Max, maximum; Mdn, median; Min, minimum.

Within the ID group, there were no significant velocity or APD differences in the ‘alone’ condition (p=0.22 and 0.67, respectively) nor the ‘pacer’ condition (p=0.48 and 0.11, respectively) between the first five (with feedback) and the last 2 min (without feedback). The stepwise multiple comparison analyses confirmed that there were no significant velocity and APD differences between any of the first five (with verbal feedback) with any of the last 2 min (without verbal feedback) in both conditions (aim 2, whether verbal feedback could facilitate the ability of people with ID to maintain a submaximal velocity).

**Figure 3:**
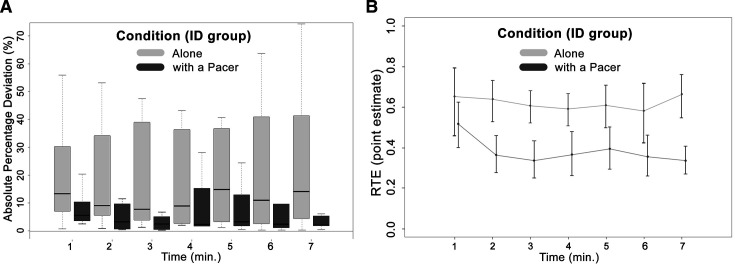
Box plots with the APD differences (A) and the relative effects (+95% CIs) (B) of the ID group at the ‘alone’ and the ‘with a pacer’ condition. APD, absolute percentage deviation; ID, intellectual disability; RTE, relative effect.

The third aim’s analyses (explore if the introduction of a pacer or cycling alone with auditory feedback can help participants with ID to keep a steady pace) did not reveal significant (main nor interaction effects) differences between the participants’ velocity and the targeted velocity of ID group in the ‘alone’ nor in the ‘pacer’ condition. The velocity’s np-CVs were significantly higher in the ‘pacer’ condition (p=0.02; see table 1 and figure 3 for the velocity variability per minute of the ID group in both conditions). The APD was significantly lower in participants with ID when they were cycling together with a pacer than alone, *F*(1, ∞)=45.40, p<0.001 statistics, respectively (between-factor main effect; aim 3). Pairwise comparisons revealed significant differences between conditions (p<0.001) at each time point of the trials except for the first minute (p=0.09). In both conditions, the APD did not significantly change throughout the trial (see table 2 for the descriptive statistics and figure 3 for the APD differences between the ‘alone’ and the ‘pacer’ condition).

## Discussion

The current study aimed to explore the differences in the ability of people with and without ID to maintain a preplanned steady pace (aim 1), if verbal feedback could facilitate the ability of people with ID to maintain a submaximal velocity (aim 2), and if contextual factors like the visual (guidance from an avatar/pacer) or auditory feedback (cycling alone) can help participants with ID to keep a steady pace during a cycling activity (aim 3). The results supported our first hypothesis, as they revealed that people with ID have an impaired ability to maintain a preplanned submaximal cycling velocity and have higher velocity variability than people without ID. This indicates that the role of ID and cognition in exercise regulation is related to the self-regulatory limitations of people with ID (‘functions’ according to the ICF).^3 16^ People with ID are dealing with impaired attention skills^33^ which impact their ability to appropriately maintain on-task behaviours and monitor their actions.^34^ Their inadequate strategies to maintain self-control and low inhibition levels^35^ could also justify their inability to maintain a preplanned, steady pace.

Due to the impaired pacing skills of people with ID, it is important to explore how we can improve and support the pacing behaviour of this population.^16^ The findings do not support our hypothesis (verbal feedback will facilitate the participants’ pacing performance) as the researchers’ verbal feedback was a contextual factor that did not improve the ability of this population to maintain a steady pace. Even if coaches could facilitate the pacing behaviour of their athletes,^15 36^ providing verbal feedback to people with ID seems more complicated. Due to their impaired judging and communication abilities, they may misunderstand instructions and respond differently to verbal cues.^37^ Moreover, due to the multitasking and multisensory (combined visual/auditory) deficits of people with ID,^38 39^ the coupling of visual and verbal feedback (as well as the coupling of visual and auditory feedback at the ‘alone’ condition) may be more challenging for this population. It cannot sufficiently reduce the cognitive load of the cycling trial.

As the verbal feedback seems challenging and did not improve the pacing skills of people with ID, it is important to explore other, less cognitive-demanding contextual factors to further assist their pacing behaviour and associated performance. The findings supported our hypothesis (the presence of a pacer will facilitate the pacing performance of the participants), as the presence of a pacer improved the ability of participants with ID to maintain a preplanned submaximal cycling velocity compared with the ‘alone’ condition (auditory feedback). This could indicate that cycling with a pacer (visual feedback only) is a less cognitively demanding strategy based mainly on intuitive than deliberate decision-making.^40^ During this process, the coupling of perception (eg, the position of the pacer) and action (eg, velocity adaptation) could facilitate a more appropriate pacing behaviour.^7 21^ More specifically, it seems that the pacers act as social affordances that alter the pacing actions of the cyclists with ID by keeping people with ID focused, motivated and engaged.^13 21^ In addition, pacers could be considered as the visual representation of the trial’s goal (maintain a submaximal velocity) and provide feedback to the participants about their pacing behaviour and enhance athletes’ self-control ability.^15 41^

This study revealed that people with ID have an impaired ability to maintain a preplanned submaximal cycling velocity, and the presence of a pacer, but not verbal feedback (nor auditory feedback), is a contextual factor that improved the ability of participants with ID to maintain a targeted submaximal cycling velocity. As it is critical to explore ‘how’ to include people with ID in sport environment, this finding is something that coaches should consider, as it could make participation of people with ID in exercise and sports less challenging.^42^ Coaches need to have the appropriate knowledge of the functions that influence their athletes’ functioning.^3^ Moreover, they should be aware of their athletes’needs and adaptations to sufficiently interact with them in exercise settings and offer them mainstreamed exercise and sports opportunities.^43^ For instance, due to communication deficits, verbal feedback may not be the most beneficial way to give directions and instructions. The coupling with a peer, however, could be a contextual factor that helps them feel more included in mainstream sports activities and build rapport with other exercisers. As people judge competence, evaluate success and failure, and continue engaging in exercise and sports based on social comparisons,^15^ peers can offer positive sport and exercise participation experiences and facilitate the sport-related skills acquisition of people with ID during submaximal exercise activities.^43^ However, it is worth mentioning that to promote equity in sports, it is crucial to provide a range of sport opportunities for people with and without ID, including regular sports with no modifications as well as segregated sport activities.^42 44^

It is worth mentioning that future studies should pay more attention to the heterogeneity of the groups, especially of the ID group. This includes individual differences in age, physical abilities and sports preferences. One approach could be to select a sample of participants who are only involved in sports where pacing is critical, such as distance running, cycling and/or swimming.^4 41 45^ This would provide a more comparable group for analysis and potentially increase the generalisability of the findings. It is also important to consider the ecological validity of the simulation itself, which refers to how closely the simulated environment matches real-world situations.^46^ For example, the cycling simulation on a stationary bike in a laboratory may not accurately reflect the real-world experience of a cycling training session and/or may not fully capture the complex and unpredictable nature of competitive sports.^47^ Therefore, it is important to interpret the findings critically and encourage future research focusing on real-world environments to more accurately capture the challenges and demands of sports participation and performance.^47^

### Conclusions

This study confirmed the role of ID in exercise regulation by showing that people with ID have an impaired self-regulatory ability to maintain a preplanned submaximal steady pace. Moreover, verbal and auditory feedback may not be appropriate to improve the self-regulatory and pacing skills of people with ID. However, introducing a social affordance (another cyclist) is an intuitive stimulus that provides direct pacing and performance feedback in visual stimuli, positively influences the pacing skills of people with ID and creates an inclusive atmosphere that promotes exercise engagement. The study provided deeper insights into our understanding of exercise behaviour and the role of cognition and self-regulation. Knowledge gained from this study can be used by coaches who want to coach people with ID effectively and who want to offer inclusive exercise opportunities.^48^

### Research reporting guideline

This paper used the Transparent Reporting of Evaluations with Nonrandomised Designs (TREND) guidelines to enhance its reporting quality.^49^

## Data Availability

Data are available in a public, open access repository.
